# Effect of Intermetallics and Drill Materials on the Machinability of Al-Si Cast Alloys

**DOI:** 10.3390/ma15030916

**Published:** 2022-01-25

**Authors:** Yasser Zedan, Guillermo H. Garza-Elizondo, Mahmoud Tash, Agnes M. Samuel, Herbert W. Doty, Victor Songmene, Fawzy Hosny Samuel

**Affiliations:** 1 École de Technologie Supérieure, Département de Génie Mécanique, Montréal, QC H3C 1K3, Canada; yasser.zedan@etsmtl.ca (Y.Z.); victor.songmene@etsmtl.ca (V.S.); 2 Département des Sciences Appliquées, Université du Québec à Chicoutimi, Chicoutimi, QC G7H 2B1, Canada; memogarzae@gmail.com (G.H.G.-E.); fawzy-hosny.samuel@etsmtl.ca (F.H.S.); 3 Department of Metallurgical Engineering Program, Cairo University, Cairo 12613, Egypt; mahmoud_tash1@yahoo.com; 4 General Motors Global Technology Center, Warren, MI 48093-2350, USA; herb.doty@gm.com

**Keywords:** drilling, cutting forces, cutting moments, intermetallics, free cutting elements, tool life

## Abstract

The present study was conducted on the machinability of 396 alloy (containing approximately 11% Si) and B319.2 alloy mainly to emphasize the effects of Fe-intermetallics, i.e., *α*-Fe, *β*-Fe, and sludge. The results demonstrate that the presence of *sludge* in the form of hard spots has a significant effect on cutting forces and tool life, in that it decreases drill life by 50% compared to the base alloy. The formation of the *α*-Fe phase in the M1 base alloy has a beneficial effect on tool life in that this alloy produces the highest number of holes drilled compared to alloys containing *sludge* or *β*-Fe; this result may be explained by the fact that the formation of the *α*-Fe intermetallic, with its rounded Chinese script morphology and its presence within *α*-Al dendrites, is expected to improve matrix homogeneity via hardening of the soft *α*-Al dendrites. Increasing the Fe-content from 0.5% to 1% in the 396-T6 alloy containing 0.5% Mn produces a distinct improvement in alloy machinability in terms of cutting force and tool life. The addition of Fe and/or Mn appears to have no discernible effect on the build-up edge area (BUE) and chip shape.

## 1. Introduction

**Figure 1:**
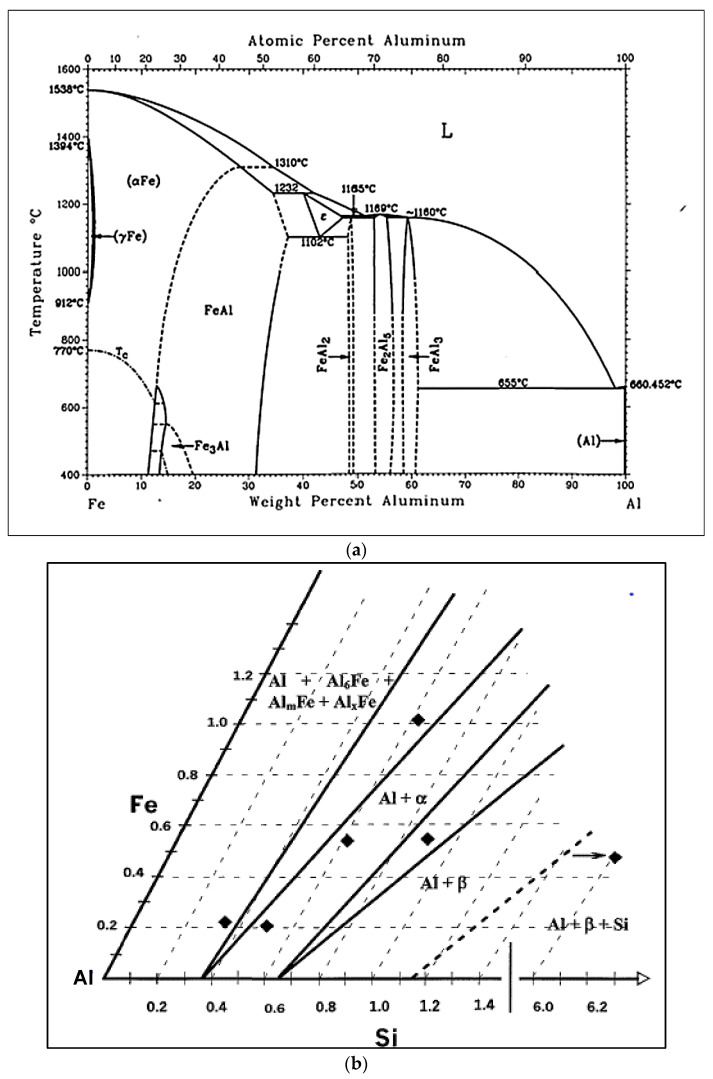
(**a**) Binary Al-Fe equilibrium phase diagram [1]. (**b**) Al corner of Al-Fe-Si phase diagram [2].

The 319 and 396 cast aluminum alloys are typically used for the production of aircraft pump parts, automotive transmission cases, aircraft fittings and controls, water-cooled cylinder blocks, and numerous other applications. Improvements to these alloys in terms of their mechanical properties are carried out through melt treatments such as grain refining and modification. Liquid aluminum is capable of dissolving iron from unprotected steel tools and furnace equipment. Iron levels can reach 2 wt% at a normal melt temperature of ~700 °C; it should be noted that an Al-Fe eutectic exists at 1.7% Fe at 655 °C, as shown in Figure 1a [1], whereas Figure 1b shows the iron intermetallic phases in the Al corner of the Al-Si-Fe system [2]. The negative effects of iron in aluminum-silicon alloys can be minimized or overcome by applying any of the following techniques [3]:

- rapid solidification,
- addition of neutralizing elements such as Mn, Cr, Be, and Ca,
- melt superheating,
- strontium modification, and
- non-equilibrium solution heat treatment.

Iron combines with other elements to form a variety of intermetallic compounds depending on the composition of the alloys; although iron intermetallics improve strength and hardness, they tend to reduce ductility. Iron also tends to make the material brittle, and consequently it improves the machinability in terms of the cutting force [4,5]. Jorstad [6] found that intermetallic phases that are formed out of heavy elements such as iron are liable to lead to a substantial reduction in tool life, although they have a negligible effect on tool-edge build-up. A number of Fe-rich intermetallic phases, including *α* Al_15_(Fe,Mn,)_3_Si_2_, *β*(Al_5_FeSi), *π*(Al_8_Mg_3_FeSi_6_), and *δ*(Al_4_FeSi_2_), have been identified in Al-Si casting alloys [7,8]. 

In near-eutectic alloys with iron concentrations of between 0.5 and 1.2 wt%, primary crystals of Al_5_FeSi (*β*-Fe) may appear under normal melt and casting conditions, and these crystals are needle-shaped. These long iron-rich needles form during solidification and are usually associated with the shrinkage voids present in the solidified material [9]. Since the Al_15_(Fe,Mn,Cr)_3_Si_2_ (*α*-Fe) phase is dendritic, it is therefore not so detrimental from the viewpoint of the machinability characteristics of the alloy, given that its *Chinese-script* morphology diminishes the shrinkage effect resulting from the concentration of stresses [10]. 

Although the *α*-Fe compound is not formed under normal casting conditions, it may be stabilized by means of the addition of certain elements, such as Mn, Cr, or Co, which substitute for iron in the crystalline structure of the intermetallic compound. Rare earth elements are also added to refine *α*-Fe compounds in cast aluminum alloys [11]. The most adequate Fe-to-Mn ratio for stabilizing the *α*-AlFeSi phase is 2:1, with iron, manganese, and chromium tending to segregate towards the bottom of the molten aluminum and holding furnaces, thereby forming solid particles of *α*-phase(Al_15_(Fe,Mn,Cr)_3_Si_2_). This phase is denser than molten aluminum and forms solid particles of *sludge* [6]. One of the approaches suitable for improving machinability is the use of free-cutting elements to improve the machinability of any given material because they provide a smooth surface, cause less tool wear, and produce chips which are more easily breakable [12]:

It should be mentioned here that the pertinent machinability criteria relate to force and moment as well as to tool life, chip configuration, and build-up edge (BUE) evolution. In keeping with these aims, the objectives of this study will cover the following:

1. specific T6 heat treatments selected to establish the hardness level for the alloys studied within the range of 110 ± 10 BHN,
2. effects of iron intermetallics (*α*-Fe, *β*-Fe, and sludge), on the machining of modified and grain refined B319.2 and 396-T6 alloys,
3. effects of free-cutting elements, such as Sn on tool life, chip shape, and cutting force and moment of the B319.2 and 396-T6 alloys, and
4. role of the type of drilling tools.

## 2. Experimental Procedure

The 396 and B319.2 base alloys used in this study were supplied in the form of 12.5 kg ingots. The chemical composition of the ingots is shown in Table 1. Eight alloys were prepared using the 396 alloy, they were then classified into three groups according to the additives used, namely, Fe-intermetallic elements, matrix-hardening elements, and free cutting elements. Three alloys of the B319.2 commercial Al-Si alloy were used to investigate the role of free-cutting elements, namely, Sn in connection with the machinability performance of these alloys. The as-received 396 and B319.2 ingots were cut into smaller pieces, dried, and melted in charges of 100 kg each, for the preparation of the various alloy compositions. Melting was carried out in a SiC crucible of 120-kg capacity, using an electrical resistance furnace in which the melting temperature was maintained at 750 ± 5 °C. All the alloys were grain-refined by adding 0.12% Ti as Al-5% Ti-1% B in rod form and modified by adding 200 ppm Sr in the form of an Al-10% Sr master alloy by means of a perforated graphite bell. Iron and Mn were added in the form of Al-25% Fe and Al-25% Mn master alloy, respectively, whereas Sn was added as pure metal.

The melt was degassed by means of a rotary graphite impeller rotating at ~150 rpm for ~15–20 min, using pure dry argon at a gas flow rate of 0.4 m^3^/h, to ensure homogenous mixing of the additives. It should be noted that, for all the castings involved, the humidity level was between 18 and 20%. The surface oxides and inclusions were skimmed off thoroughly prior to pouring. Two sets of castings were prepared. Samplings for chemical analysis were also taken concurrently for each melt condition; the chemical analysis was carried out at the GM facilities in Milford, NH, using a Spectrolab Jr CCD Spark Analyzer (Spectro Analytical Instruments Inc., Mahwah, NJ, USA). 

**Figure 2:**
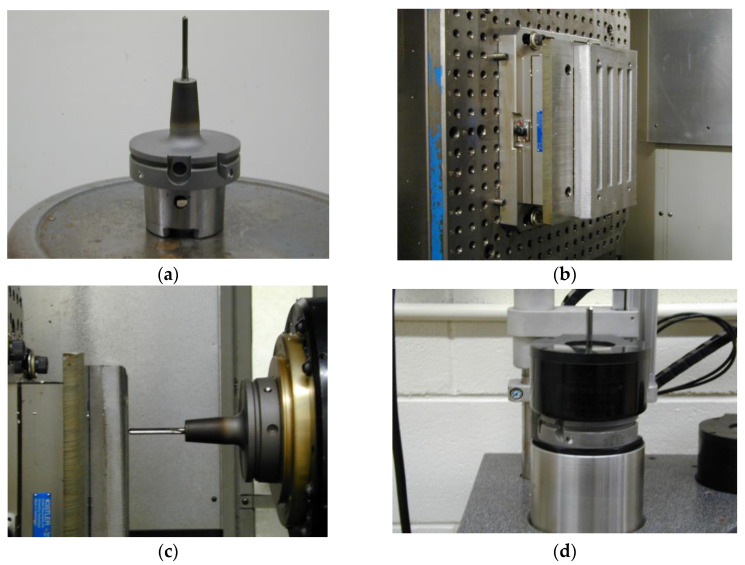

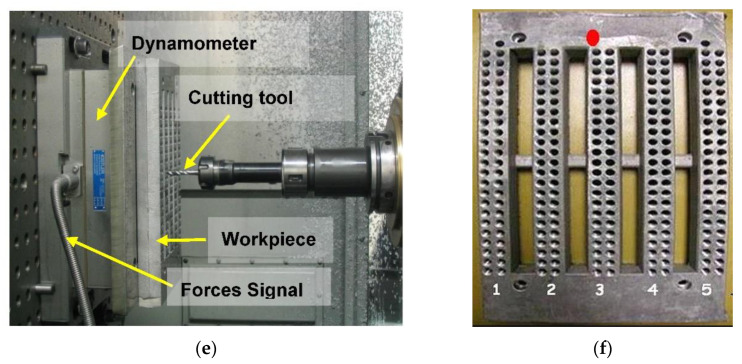
Drilling and tapping experimental set-up: (**a**) tool set-up; (**b**) sample and dynamometer cable set-up; (**c**) horizontal machining set-up; (**d**) tool removal during cooling cycle; (**e**) photograph showing experimental set-up; and (**f**) drilled Al cast block.

The castings were divided into two sets. The averages obtained for all the different chemical analysis samples and the respective codes for the various castings prepared from the base alloys 396 and B319.2 are listed in Table 2 and Table 3. For the purpose of comparison, limited tests on drilling and tapping forces were carried on a third series, Table 4, where the amount of Mg content in the 319-type alloys was varied, i.e., A319 and B319.2 with two levels of Fe. The melt was poured into a graphite-coated waffle-plate metallic mold which had been preheated to 450 °C to prepare the castings for machinability studies; eighteen machinability test blocks were cast. The test blocks were subsequently cut from each casting and then machined to the final testing form having the overall dimensions of 300 mm length, 175 mm width, and 30 mm thickness, with five ribs each approximately 25 mm wide, separated by gaps of 16 mm, as shown in Figure 2.

The heat treatments were selected in such a way as to establish the hardness level as a common factor for all the alloys studied within the range of 110 ± 10 BHN. This range is the one most frequently used in the majority of commercial applications for aluminum alloys. It was thus possible to carry out solution heat treatments at 490 °C/8 h for the 396 and B319.2 alloys. The solution heat-treated samples were quenched in warm water at 65 °C, followed by artificial aging at 200 °C for 5 h for the 396 and B319.2 alloys for the first set, and 5 h at 180 °C for the second set of alloys, i.e., T6 tempered. The heat treatments were carried out in a forced-air Blue M electric furnace equipped with a programmable temperature controller accurate to within ±2 °C for both the solution and the aging treatments. Heat treatment of the A356 alloy was the same except that, in this case, the solutionizing temperature was 540 °C. 

Samples measuring 25 mm × 25 mm for metallographic examination were sectioned from the machinability test sample. The microstructure was examined with an Olympus PMG3 optical microscope. The eutectic silicon-particle characteristics, including particle area, length, roundness (%), aspect ratio, and density (particles/mm^2^) were measured and quantified using a Clemex image analyzer system in conjunction with the optical microscope. For each sample, 50 fields were examined at a magnification 500×, in such a way as to cover the entire sample surface in a systematic manner. 

Hardness measurements were thus carried out on the heat-treated machinability test blocks employing a Brinell hardness tester, using a steel ball of 10 mm diameter and a load of 500 kg applied for 30 s. The average hardness of the four blocks was then obtained to represent the hardness value for each alloy condition. Tensile test bars were produced by pouring the degassed molten metal at 450 °C into a preheated permanent steel mold, type ASTM B-108; each mold casting provided two tensile bars, each with a gauge length of 50 mm and a cross-sectional diameter of 12.7 mm. Five bars were prepared for each alloy composition. The test bars were solution heat-treated at 490 °C for 8h, then quenched in warm water at 65 °C, followed by artificial aging at 200 °C for 5 h (i.e., T6 tempered). The heat-treated test bars were pulled to fracture at room temperature at a strain rate of 4 × 10^−4^/s using a Servo-hydraulic MTS Mechanical Testing machine. 

Drilling and tapping tests were carried out using a Makino A88E high-speed 5-axis, high-power horizontal machining center with a maximum power reading of 40 HP (30 kW) and a maximum rotation speed of 18,000 rpm. The experimental set-up consisted of an A88E machine, a dynamometer with four sensors, charge amplifiers, and an A/D converter; this set-up was applied for the online measurement of drilling/tapping forces and moments. Additionally, a toolmaker’s microscope was used for observing the BUE formation and tool-wear characteristics. 

For the first set of castings (Table 2), the drills were made of uncoated ISO K20 carbide or RT 150 GG according to Gühring specifications. A straight flute and coolant-fed carbide “G” drill with an average diameter of 6.5 mm, 2-flute, 120° point angle and 90 mm total length was used to drill two rows of blind holes in each rib, i.e., 36 holes per rib of the waffle-plate, producing 180 holes per casting block. Titanium-nitride coated HSS cutting taps, M8*1.25-6H, with three spiral flutes were used subsequently for the tapping process. Tapping was carried out for the drilled holes of the full plate immediately after drilling. Schematic representations of the carbide drill and HSS tap, together with the terms used in describing their geometry are presented in Ref. [13]. 

Four special drills were used when working with the aluminum alloys described in Table 3. The drills chosen for use were:

- solid carbide drill (Gühring 768-Bright),
- special solid carbide drill (Gühring 5512-Firex),
- cobalt grade drill (Kennametal B411A06500),
- solid carbide high precision drill (Mapal Giga-Drill M2195-0650).

All alloy conditions were tested under the same cutting parameters for drilling and tapping tests. The drilling tests were carried out at rotational speeds of 11,000 rpm using a feed rate of 1.117 m/min with each hole being 28.38 mm deep. The tapping tests were conducted at low cutting speeds, i.e., 400 rpm, using a feed rate of 0.56 m/min with each tapped hole being 20.76 mm deep. 

A Kistler, model 9255B, 6-component piezoelectric quartz crystal dynamometer was used during drilling and tapping tests for the online measurement of the cutting forces and moments. The measuring system using piezoelectric force differs from other methods of measurement. The cutting forces acting on the quartz crystal elements were converted to a proportional electric charge in Pico-Coulombs (PC). The output charges from the dynamometer were guided by an amplifier through the eight-core connecting cables, type 1677A5/16779A5. The charge amplifier converted these charges into proportional voltage signals which were subsequently converted into force signals by an analog-to-digital (A/D) converter using a data acquisition system. These force signals were independently monitored and recorded for each test block in the LabVIEW program whereas Cut Pro 8.0 software was used for the cutting force measurements. 

The chips produced during the drilling tests were collected for the purposes of examining their size and shape after drilling each test block. Chip size was studied using the number of chips-per-gm criterion. The build-up-edge (BUE) width of the drill was observed at the end of each sample casting test while the failure of the first and second teeth of the tap was also checked using a toolmaker’s microscope (TM-505 type) at a magnification of 30×.

## 3. Results and Discussion

### Microstructural Characterization

Table 5 summarizes the silicon particle characteristics obtained from quantified measurements of the alloys investigated as shown in Table 2. The morphology of eutectic Si plays a vital role in determining the machinability characteristics of cast Al-Si near-eutectic alloys. A fine, well-modified eutectic silicon structure is far less detrimental to tool life than heavy element intermetallic phases; tool life, will however, decrease in the presence of a coarse eutectic silicon structure. The Si particles were present in the form of coarse acicular plates with an aspect ratio of 3.51 in the T6 heat-treated condition for the non-modified experimental M0 alloy. The addition of 200 ppm of Sr transformed the morphology of the Si particles from an acicular form to a fibrous one, as may be observed in the M1 alloy which has an aspect ratio of 1.77; this addition also increases the roundness ratio from 32.8% to 70%. 

The average Si particle length decreased from 20.80 μm in the M0 alloy to 2.96 μm in the M1 alloy, while the average area decreased from 65.70 μm^2^ to 4.30 μm^2^, i.e., by about 86% and 93%, respectively. As a result of the decrease in the size of the particles, the density of the Si particle increased from 2604 to 28,000 particles/mm^2^, implying that the eutectic Si particles were fibrous and finely divided in the presence of Sr. It is also noted that the increase in the level of Fe and/or Mn in the M3 and M4 alloys, respectively, had only a slight influence on the values of the average Si particle including area, length, roundness, aspect ratio, and density as compared to the M1 alloy, provided in Table 5 for the first set of alloys as shown in Table 2.

The percentage of Si in Al-Si alloys together with the shape and distribution of the silicon particles all play an important role in determining the machinability characteristics of cast Al-Si alloys, as was observed by examining the microstructures of the modified grain-refined and T6 heat-treated G2 and G12 alloys, containing Al-10.8% Si and Al-7.5% Si. Table 6 summarizes the eutectic Si-particle characteristics obtained from quantitative measurements of the second set of alloys (Table 3) investigated with respect to additions. It will be observed that the addition of 0.15% Sn to the G2 and G12 alloys leads to a slight coarsening of the eutectic Si particles, as may be observed from the data listed in Table 6.

**Figure 3:**
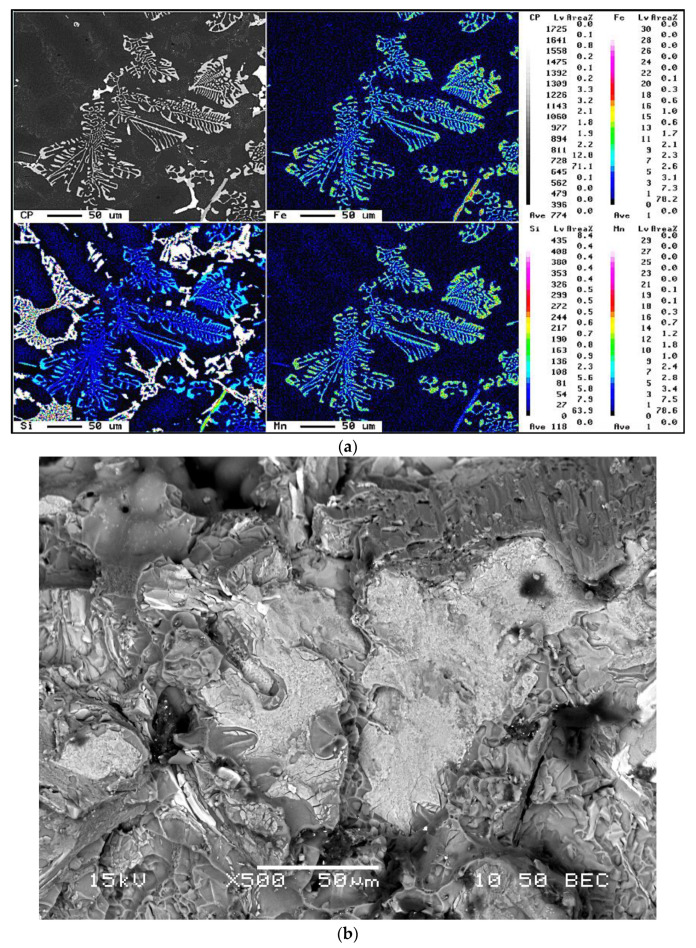

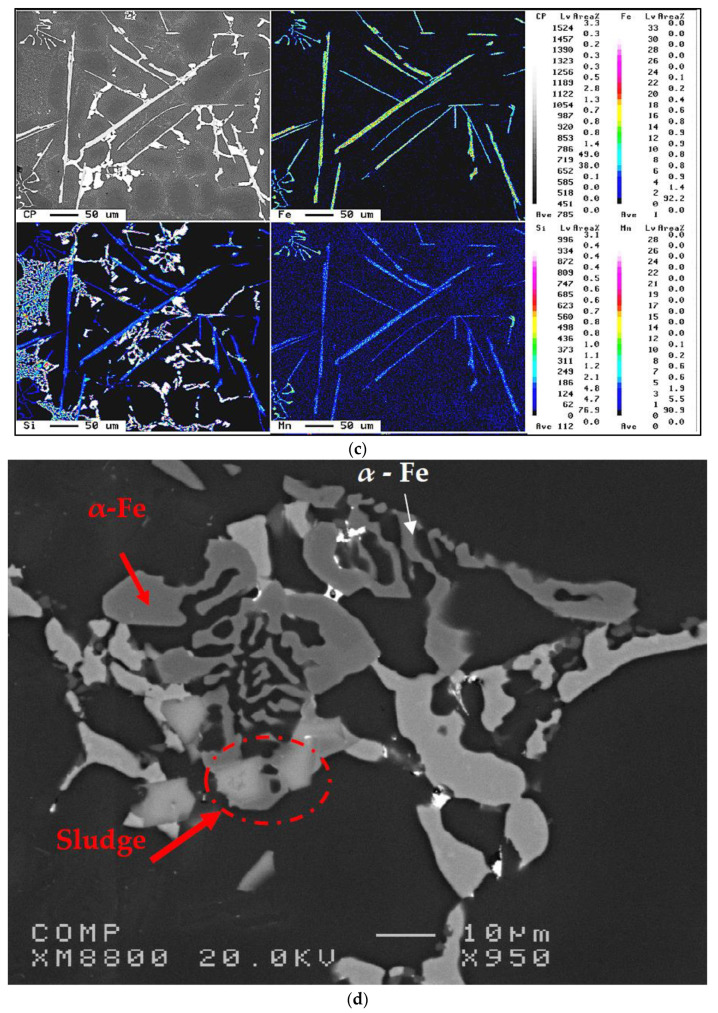

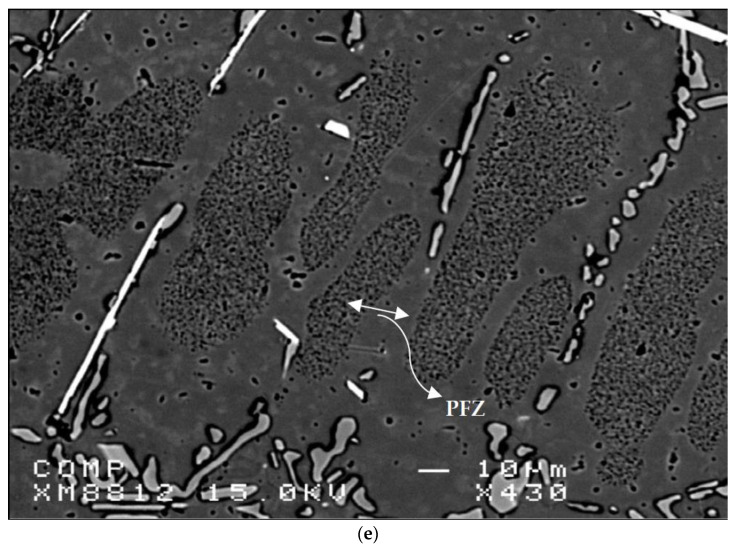
(**a**) Elements distribution in α-Fe phase; (**b**) Fracture surface showing *sludge* particles observed in the Sr-modified and grain-refined M3 alloy in the as-cast condition; (**c**) Elements distribution in the β-Fe phase; (**d**) Backscattered electron image showing the *sludge* particles observed in the Sr-modified and grain-refined G3 alloy; (**e**) Backscattered image taken from etched A356-T6 (180 °C/4 h) showing precipitate-free zones (PFZs) in the vicinity of the β-Fe intermetallics—note the fragmentation of the β-platelets during the solutionizing treatment.

The morphology and size of the eutectic Si particles, together with the precipitation-hardening phases during heat treatment, all had an important influence on machining properties. It was found that the addition of Sn elicited a similar behavior from both the 396 and B319.2 alloys, where the addition of 0.15% Sn had little influence on the eutectic Si particles. The size, shape, and distribution of free-machining particles, in this case Sn, are of major importance in the machining of Al-Si casting alloys. The iron precipitates in the form of a coarse *α*-Fe phase, which is always seen to occur within the *α*-Al dendrites, indicating that it corresponds to a predendritic reaction, and thus tends to nucleate in the liquid alloy prior to solidification. The formation of the *α*-Fe phase reduces the possibility of the formation of the *β*-Fe phase [14]. As shown in Figure 3a,b the coarse *α*-script particles were always seen to occur within *α*-Al dendrites in the M1 alloy indicating that they corresponded to a pre-dendritic reaction and tended to nucleate in the liquid alloy prior to solidification. The large size of these particles indicates the existence of a high diffusion rate of Fe occurring at high melt temperatures [15].

The *α*-Fe phase may precipitate either in the form of the *Chinese-script* phase or else as compact polygonal or star-like particles termed *sludge* with a composition close to that of the *α*-Fe phase. This *sludge* precipitation depends upon the levels of Fe, Mn, and Cr present in the alloy, and the processing parameters which include melt holding temperature, pouring temperature, melt additives, and cooling conditions. In this study, when the iron and manganese levels were increased from ~0.5 wt% in the M1 alloy to ~0.78 wt% in the M3 alloy, the predominant shape of the Al(Fe,Mn)Si phase, with a composition of Al_12_(FeMnCu)_3_Si_2_ was polyhedral or star-like, as shown in Figure 3a,b. 

When the iron content increased from ~0.5 wt% in the M1 base alloy to ~1 wt% in the M4 alloy, Chinese-script *α*-Fe and platelet-like *β*-Fe compounds formed with the same level of Mn, i.e., ~0.54 wt%, as may be seen in Figure 3c. The *β*-Fe phase fragments via two mechanisms [16]: (i) splitting of the needle/platelets into two halves through the formation of longitudinal cracks, which was enhanced greatly by the brittle nature of the *β*-Fe phase, and (ii) fragmentation through Si rejection. These observations confirm the findings of Villeneuve and Samuel on the fragmentation and dissolution of the *β*-Fe phase during the solution heat-treatment of Al-13% Si containing different levels of iron at 540 °C [17].

Figure 3d shows the microstructure of the G3 alloy in which the *α*-Fe phase appeared in the form of small *Chinese-script* particles interspersed with *sludge* particles. The conditions which influenced the formation of the *α*-Fe phase, as opposed to the *β*-Fe phase, are still not completely understood; moreover, the formation of the *α*-Fe phase reduced the possibility of the formation of the *β*-Fe phase. The *α*-Fe phase may be precipitated either in the *Chinese-script* form, or else as compact polygonal or star-like particles termed *sludge* with a composition close to that of the *α*-Fe phase. Figure 3e is a backscattered image obtained from a Sr-modified 356 alloy sample aged at 180 °C/4 h. In this case, well defined Mg_2_Si precipitates and precipitate-free zones (PFZ) can be observed within the alloy matrix.

## 4. Hardness and Tensile Properties

With regard to machinability, it was reported that heat treatment which increases hardness would reduce the build-up edge (BUE) on the cutting tool and would improve the surface finish of the machined part. A minimum hardness value situated in the range of 90–100 BHN for an alloy casting is to be recommended since this would tend to avoid the difficulties associated with a build-up edge on the cutting tool. Thus, a specific T6 heat treatment was selected to establish the hardness level for the alloys investigated within the range of 110 ± 10 BHN.

The hardness data reported in Table 7 indicate that the decrease in the hardness value of the Sr-modified M1 alloy compared to the non-modified M0 alloy is mainly the result of changes in the morphology of the eutectic Si particles, from brittle coarse acicular plates in the M0 alloy to a rounded fibrous form, as shown in Figure 3a,b. Additionally, Sr leads to a depression in the eutectic temperature causing a shift of the eutectic point to a higher Si content, resulting in an increase in the amount of soft *α*-Al formed [18].

Table 7 provides the tensile properties including the yield strength, YS at a 0.2% offset strain, the ultimate tensile strength, UTS, and the percent elongation to fracture % El for the alloys investigated. The modified M1 alloy displayed somewhat higher YS, UTS, and % El values than the unmodified M0 alloy, because of the improved eutectic silicon phase morphology caused by the Sr modification. It should also be noted that as the percentage of Fe and/or Mn increased beyond 0.75%, the YS, UTS, and ductility decreased to a significant degree, a fact which may be attributed to the presence of the *β*-Fe phase in the structure of the M4 alloy. The high stress concentrations at the sharp edges of the *β*-phase, as well as the weak bonding between the *β*-phase and the Al matrix, enhance crack initiation and thus decrease the ductility of this alloy [19]. Table 8 provides the relevant data relating to T6 heat-treated mechanical properties; these include the hardness (BHN), yield strength at 0.2% offset strain (YS), ultimate tensile strength (UTS), and percent elongation (% El) of the 396 and B319.2 alloys for the second set of alloys. 

## 5. Machinability Evaluation

### 5.1. First Set of Alloys

The upcoming sections will discuss the machinability behavior of the alloy conditions investigated in this work with respect to the total cutting force and moment, tool life expressed as the number of holes drilled/tapped up to the point of tool breakage, chip configuration, and build-up edge (BUE) evolution. The drilling and tapping tests were carried out under fixed machining conditions of speed, feed, depth of hole drilled/tapped, tool geometry, tool material, and coolant.

**Figure 4:**
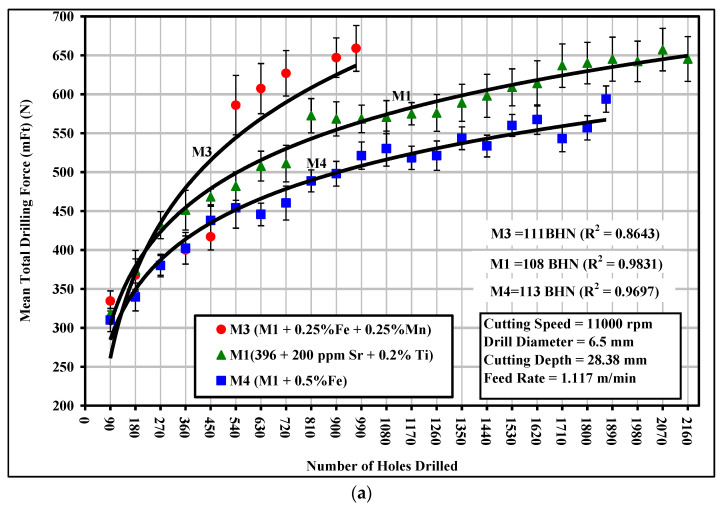

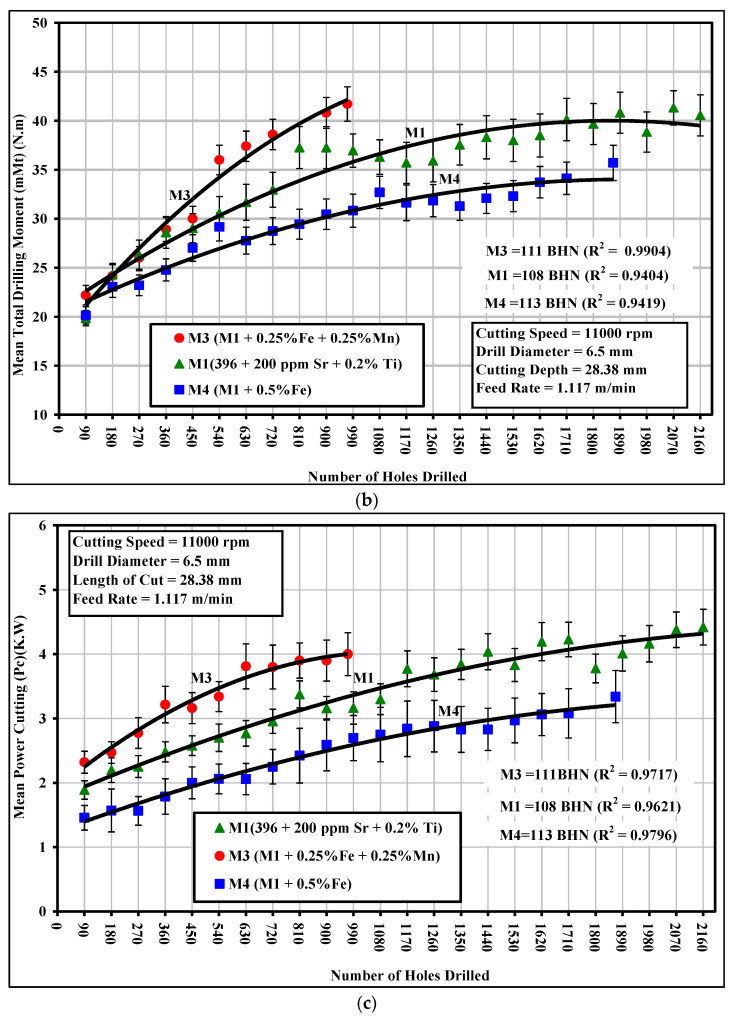
Effects of Fe-intermetallics on the machinability of M1, M3, and M4 alloys in terms of (**a**) mean total drilling force; (**b**) mean total drilling moment; and (**c**) mean power cutting required for drilling 90 holes.

The addition of 0.25% Fe and 0.25% Mn to the M1 base alloy, producing the M3 alloy which contained *sludge* as a predominant phase, led to an extremely rapid increase in the total drilling force from 334.48 N after drilling 90 holes to 586 N after drilling only 540 holes, with a corresponding increase of 75% over the evaluation period of 540 holes; similar behavior was also observed with regard to the total drilling moment and power, as shown in Figure 4. This significant increase in cutting force may be explained by the formation of the hard, complex, intermetallic sludge phase as the predominant phase in the M3 alloy. The sludge may be seen clearly in the backscattered image and optical micrograph shown earlier in Figure 3. It has also been reported that the hardness of sludge usually lies between 500 and 900 BHN, as compared to a matrix hardness of 108 BHN for the 396 alloys [20]. The sludge phase may act as an abrasive in an otherwise relatively soft matrix and it is capable of causing excessive tool wear, thereby increasing the cutting force and moment. 

The addition of 0.5% Fe to the M1 base alloy, thereby producing the M4 alloy which contained a relatively thin form of *β*-Fe as the predominant phase, resulted in a considerable improvement in alloy machinability through (i) a lowering of the drilling force by 10%, ranging from 6% to 14%, (ii) a lowering of the drilling moment by 14%, ranging from 9% to 20%, and (iii) a decreasing of the drilling power by 24%, ranging from 15% to 30%, over the evaluation period of 1867 holes compared to the M1 alloy, as shown in Figure 4a–c. The preceding observations may be understood in the light of the effects which the presence of the *β*-Fe phase is known to have on ductility. As was listed earlier in Table 6, the ductility of the M4 alloy decreased noticeably by 50%, compared to the M1 alloy. The lower ductility value displayed by the *β*-Fe-containing M4 alloy lessened the drill-chip friction which in turn decreased the drilling forces, moment, and power. It has been reported by a number of observers that, as the ductility increases, plastic deformation takes place in the cutting zone and consequently the cutting resistance becomes greater, causing the cutting force to be higher [21,22].

**Figure 5:**
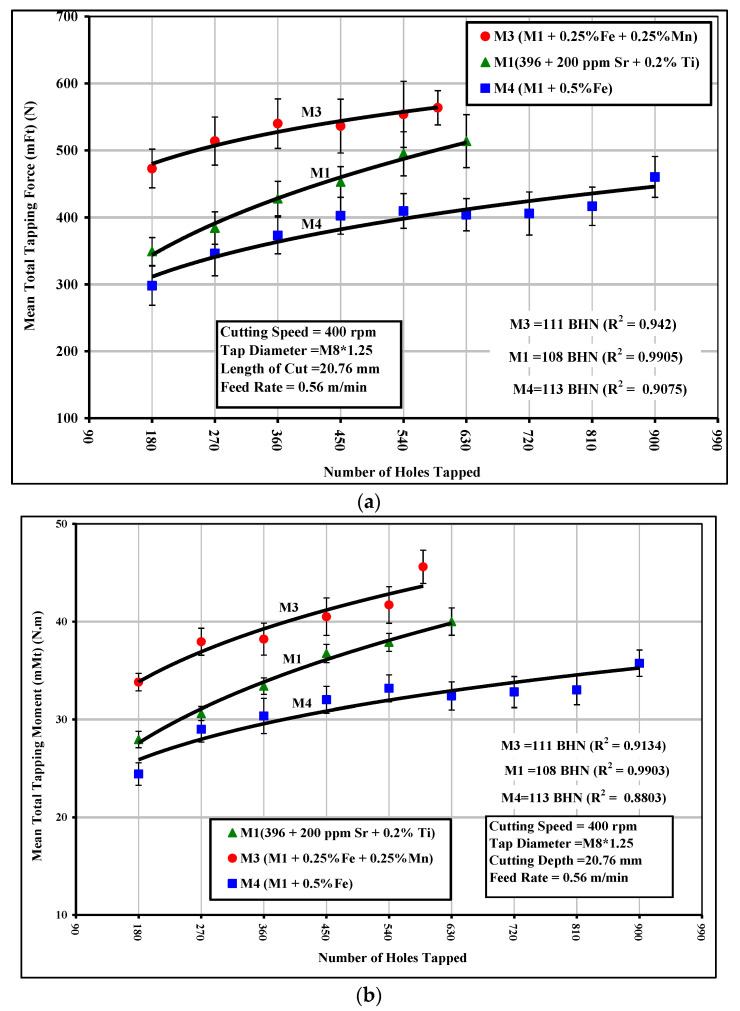
Effects of Fe-intermetallics on the machinability of M1, M3, and M4 alloys in terms of (**a**) mean total tapping force; and (**b**) mean total tapping moment required for tapping 90 holes.

In the tapping test, the mean total tapping force and mean total tapping moment were used as criteria for characterizing the alloys studied. The effects of Fe-intermetallics on the tapping force and moment for the M1, M3, and M4 alloys are shown in Figure 5a,b. It can be clearly observed that the presence of sludge as the predominant phase in the M3 alloy resulted in a noticeable increase in the tapping force and moment compared to the M1 alloy containing the *α*-Fe phase. In this regard, the M3 alloy required an average 30% higher tapping force ranging from 12% to 48% and displaying an average 15% higher tapping moment ranging from to 10% to 20% compared to the M1 alloy. It should be mentioned that HSS taps were used for the tapping test instead of a carbide drill which was used for the drilling tests; the sludging produced in this way led to such deleterious effects as hard-spot particles in the alloy structure, ultimately resulting in a significant increase in the tapping force and moment. 

It can also be observed that the thin *β*-Fe-containing M4 alloy had the same effect on the tapping force and moment as the one observed in the drilling test, since the tapping force was reduced by 11% ranging from 2% to 20% compared with the M1 alloy. The tapping moment was decreased by almost the same ratios. This reduction occurring in the tapping force/moment values of the M4 alloy may be explained in the same manner as were the effects of the low ductility values resulting from the presence of the *β*-Fe phase during the drilling tests. 

### 5.2. Second Set of Alloys 

In this section the results of the cutting forces will be presented. The addition of Sn to the 396 and B319.2 casting alloys in small amounts of ~0.15%, thereby producing the G2 and G12 alloys, significantly improved the machinability characteristics of these alloys in comparison with the G3 alloy which, with the additions of 0.25% Fe and 0.25% Mn, contained *α*-Fe and sludge intermetallic phases. It is necessary to take into account the fact that the rapid increase in the drilling force and moment in conjunction with the progress of the cutting process during the machining of the G2 alloy, which contained 10.8% Si and 2.3% Cu, may be attributed to the presence of the greater amount of silicon phase present in the alloy, which made the alloy harder than the G12 alloy which contained 7.5% Si and 3.6% Cu. This difference was reflected in the microstructure which showed much larger regions of Al-Si eutectic in the 396 alloy.

**Figure 6:**
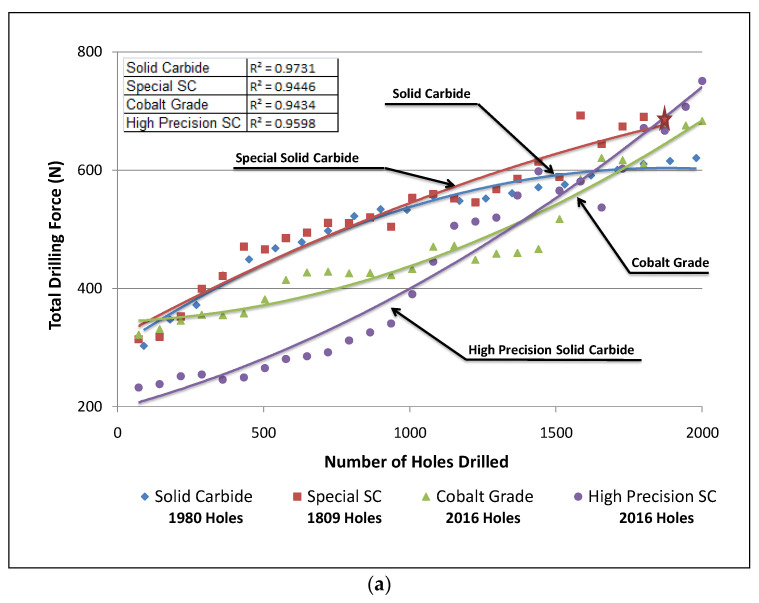

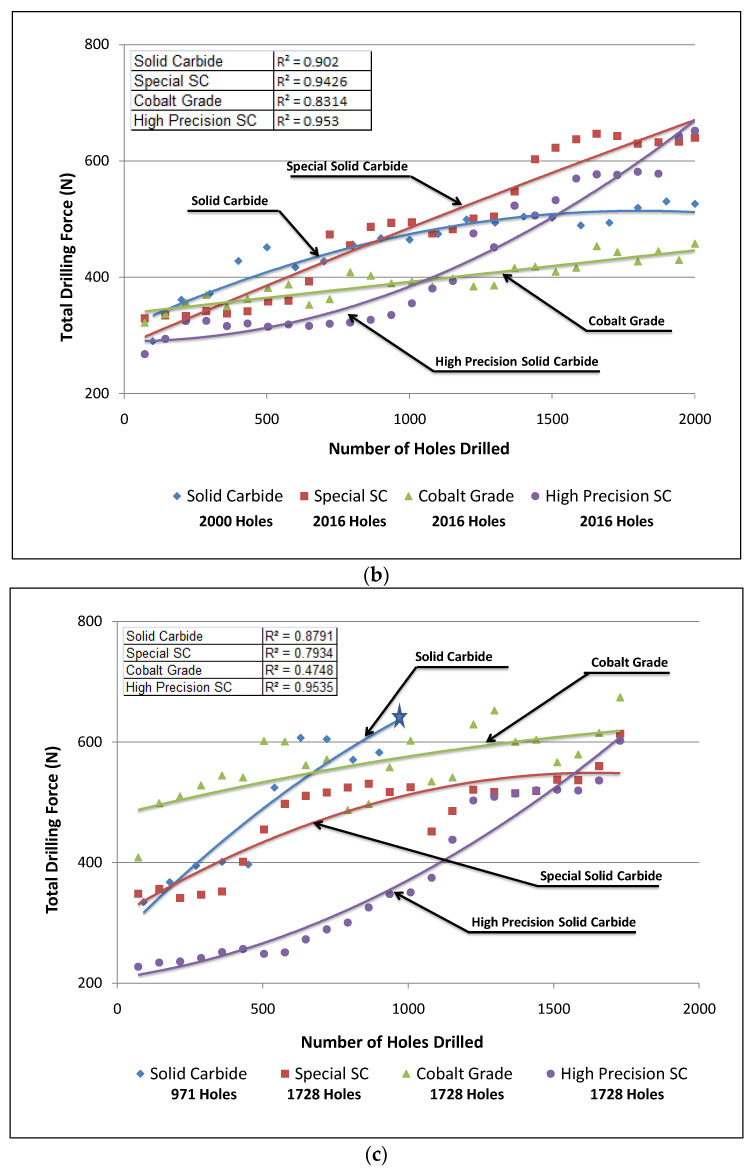
Effects of Sn on the machinability of (**a**) G2 alloy (396 + 0.15% Sn), (**b**) G12 alloy (396 + 0.15% Sn), and (**c**) G3 alloy (396 + 0.25% Fe + 0.25% Mn) in terms of total drilling force.

**Figure 7:**
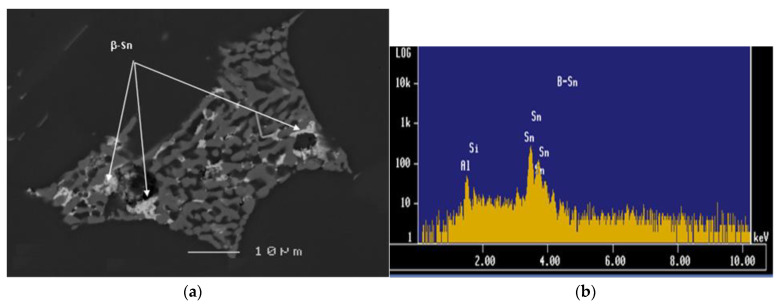
(**a**) Backscattered image obtained from the 396 alloy showing the precipitation of β-Sn, and (**b**) EDX spectrum corresponding to a β-Sn particle observed in the 396 alloy.

The 396 and B319.2 alloys yielded hardness values of 106 and 108 BHN, respectively. During drilling tests, it was found that the G2 alloy, containing 0.15% Sn and 10.8% Si, displayed the same number of holes drilled as did the G12 alloy, which contained the same percentage of Sn but a 7.5% Si content, as both alloys were observed to fulfill the test limit of obtaining 2016 drilled holes over the 14 machinability blocks tested for each alloy. The lower ductility displayed by both alloys due to the presence of Sn, decreased the drill-chip friction which in turn decreased the cutting force and moment, in comparison with the G3 alloy which contained *α*-Fe and sludge intermetallic phases, and presented a greater number of fluctuations in the results. These effects may be observed in Figure 6a,b for the G2 and G12 alloys and in Figure 6c for the G3 alloy. Figure 7 provides an example of the *β*-Sn phase observed in the as-cast 396 alloy and the corresponding EDS spectrum, confirming its presence.

Thus, it may be assumed that Sn presents a lubricating effect in the drilling process, in relation to the fact that the addition of Sn as a free-cutting element with its low melting-point allows it to act as a lubricant, thereby decreasing the friction between the chip and tool edge which tends to decrease the ductility of the material in the cutting zone, causing in turn a reduction in drilling force and moment during the cutting process.

In Figure 6, the significant increase in cutting force in the case of the G3 alloy may be explained by the formation of the hard, complex intermetallic sludge phase as the predominant phase in this alloy. It has also been reported that the microhardness of sludge usually lies between 500 and 900 BHN, as compared to a matrix hardness of 111 BHN for the G3 alloy [23,24]. The sludge phase may display behavior which is similar to that of an abrasive in an otherwise relatively soft matrix, whereby it is capable of causing excessive tool wear and resulting in an increase in the cutting force and moment. It should also be noted that the fluctuations of the total drilling force and moment values during the drilling of the G3 alloy resulted from the distribution and the size of the sludge particles within the cast alloy structure, i.e., the machinability test block itself.

The G12 alloy, which contained 7.5% Si and 0.15% Sn, revealed the lowest values of total drilling force and moment. By contrast, the presence of 0.25% Fe and 0.25% Mn in the G3 alloy caused deterioration in the drill life; it should also be noted that this alloy presented a greater number of fluctuation values in the total drilling forces and moment, with the drill breaking after only 971 holes were drilled. With regard to this observation, it is probable that the solid carbide drill became encrusted with a number of sludge intermetallic particles after which the drill breakdown occurred. The G2 alloy, containing 10.8% Si, displayed a behavior similar to that of the G12 alloy, which contained the same amount of Sn addition. 

The results of total drilling force obtained with the solid carbide drill and special solid carbide drill show that the G2 and G12 alloys, which both contained 0.15% Sn, present similar behavior with either drill type, as may be seen from Figure 6. In comparison, the G3 alloy presented greater fluctuations in drilling force and moment values with the progress of drilling. It should be noted that, in the case of the special solid carbide drill, the drill broke after drilling 1728 holes in the G2 alloy, without completing the targeted goal of 2016 holes set for this alloy, and which had been met by the other three drills in the drilling process. 

**Figure 8:**
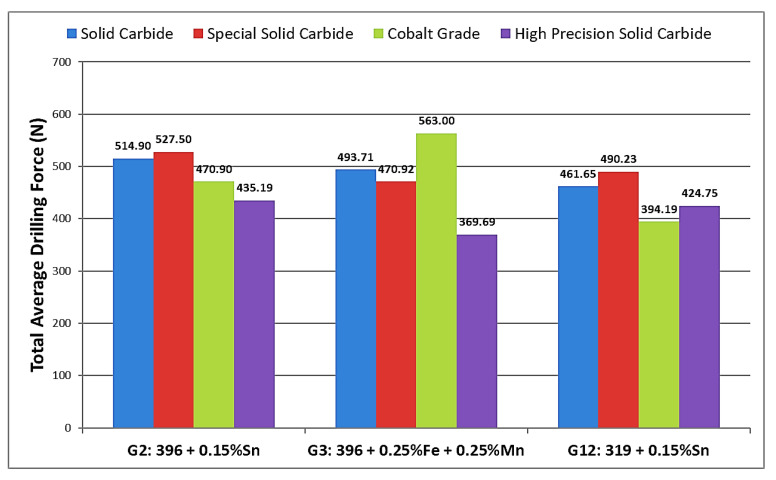
Comparison of the total average drilling forces obtained for alloys G2, G3, and G12 using different drills.

The differences in the machining behavior of the 396 and B319.2 alloys may be attributed mainly to the differences in matrix hardness, as well as to differences in the microstructural constituents resulting from alloy chemistry, additions, and heat treatment. Matrix hardness (beneficial) and alloy abrasiveness (detrimental) seem to be the real issues controlling alloy machinability. In the present case, this was investigated for the G2, G3, and G12 alloys using two Al-Si alloys, 396 and B319.2, with different Si contents, and Sn, Fe, and Mn additions, tin for its free-cutting properties, and iron and manganese for producing Fe-intermetallics, namely, *α*-Fe, *β*-Fe, and *sludge* in the alloy structure. The histograms provided in Figure 8 display the results for total drilling force for each alloy/drill combination.

### 5.3. Third Set of Alloys 

**Figure 9:**
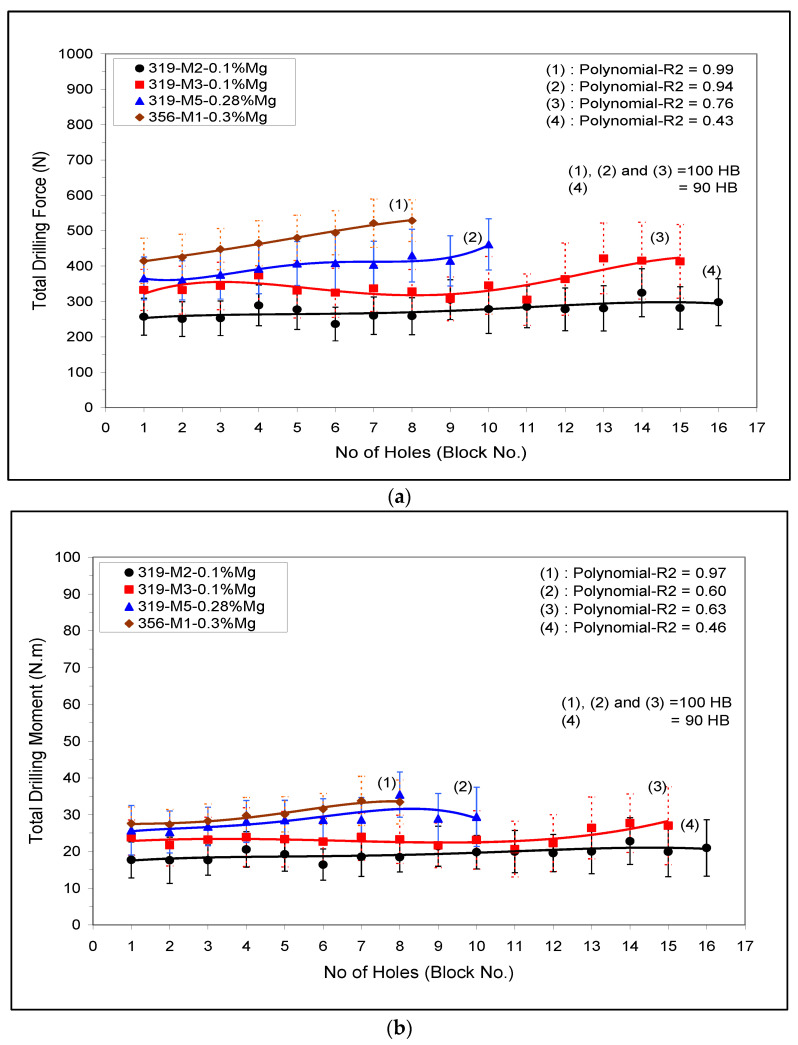
The effect of α-Fe-intermetallic volume fraction on the machinability of Sr-modified 356 and 319 alloys in terms of (**a**) total drilling force, and (**b**) total drilling moment for M1 (356), and M2, M3 and M5 (319) alloys.

The drilling and tapping (force and moment) of Sr-modified A356 (M1) and 319 (M3 and M5) alloys containing mainly α-Fe- intermetallics are shown in Figure 9a,b), respectively. The effect of both Mg content and α-Fe-intermetallic surface fraction on the cutting force and moment, viz., the M2-A319 alloy (low α-Fe-intermetallic volume fraction 2%) and M5-B319.2 alloy (high α-Fe-intermetallic volume fraction 5%) when both alloys were given the same aging treatment (220 °C/2 h), are displayed in Figure 9 (plot (4) and (2), respectively).

Drilling force and moment of the M3 and M5 conditions when aging was carried out at 180° and 220 °C for two hours are shown in Figure 9 (trend line 3 and 2, respectively). A small addition of 0.1% Mg to the M3-condition improved the alloy machinability and lowered the cutting force and moment compared with the M5 (0.28% Mg). Similar behavior was observed in the tapping results, Figure 9.

In the drilling results, it was found that the lower copper content (i.e., M1-A356 alloy) resulted in a higher cutting force compared to the M5-319 alloys with both alloys given different aging treatments but having the same hardness level (100 HB). On the other hand, the softer M1-A356 alloy gave the best results in the tapping processes, i.e., lower tapping force and moment, and hence a higher number of tapped holes than the M5-319 alloy. The higher cutting force and moment observed in the A356 alloy can be attributed to the ductility, and the presence of precipitates free zones (PFZs) as shown in Figure 3e; however, the A356 alloy gave the best results during the low speed tapping processes. 

## 6. Tool Life

### 6.1. First Set of Alloys

A comparison of the M1 and M3 drilling results, notably in terms of the number of holes drilled, revealed that the M3 alloy, which contained mainly sludge of the hard-spot type as the predominant phase, was more sensitive to tool wear than was the M1 alloy. Hence, the presence of *sludge* had a significant effect on tool life, in that it decreased drill life from 2160 holes in the M1 alloy to 971 holes in the M3 alloy, which is a corresponding decrease of 50%. From the preceding observation, it is clear that the sludge phase has high hardness values, a high melting point, and a high specific gravity compared to the matrix, and it is consequently capable of causing damage to cutting tools; this phase is liable to cause rapid tool wear and to bring on all of the attendant problems accompanying a dull tool, as was confirmed by drill wear examination using a toolmaker’s microscope.

On the other hand, the M4 alloy, containing mainly *β*-Fe as the predominant phase, displayed an increase in the length of drill life by 48% compared to the M3 alloy, showing a slightly lower number of holes drilled compared to the M1 alloy, namely 1867 holes with 2160 holes drilled in the M1 alloy. It is interesting to note that increasing the iron level from ~0.5 to 1% while maintaining the same Mn level of ~0.5% proved to be the most effective in terms of cutting force and tool life in the drilling and tapping processes. This observation may be explained by the fact that the detrimental effects of Fe may be partially neutralized by adding Mn in proportions greater than half the Fe concentration. As shown in the discussion on microstructure and illustrated in Figure 3, the fragmentation of the *β*-Fe and the production of a relatively finer *β* phase will tend to have a direct influence on increasing the homogeneity of the matrix and hence prolonging tool life as a result of solution heat treatment and the addition of Sr. This outcome was also consistent with results recorded by Komiyama et al. [25] who showed that Mn has a powerful effect on alloy properties although only at iron contents of greater than 1%. 

**Figure 10:**
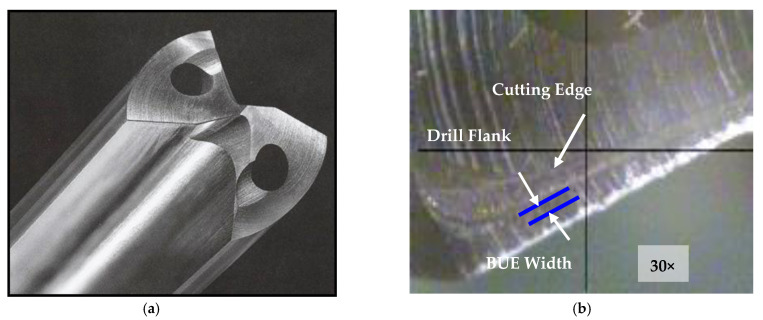

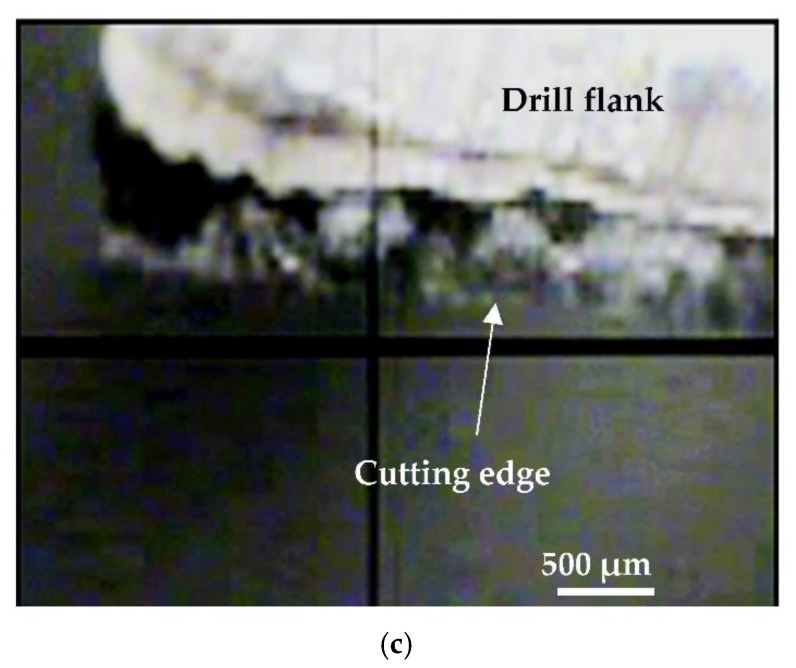
Photographs showing the progress of build-up edge (BUE) on the tip of the cutting edge (**a**) fresh drill, (**b**) M1 alloy after drilling 2070 holes, and (**c**) M3 alloy after only 900 holes due to *sludge* formation.

The tendency of each alloy to adhere and build up on the cutting edge of the tool was detected through a high magnification top-view of the projected area looking at the actual build-up on the drill; this was carried out using a toolmaker’s microscope (TM-505 type) at a magnification of 30×. Figure 10a,b) show photographs of the progress of edge build-up on the cutting-drill lip from the beginning to the end of drilling the 396 alloy casting. 

Figure 10c displays the accumulation of sludge on the edge of the cutting tool. An analysis of the edge build-up and an examination of the corresponding photographs indicate that there was little change to be seen in the width of the BUE after drilling the different numbers of holes. This fact may be attributed to the amount of the deposit, or the BUE, which gradually increased; when this exceeded a critical size, however, it separated from the cutting face and adhered to the lower surface of the chip.

**Figure 11:**
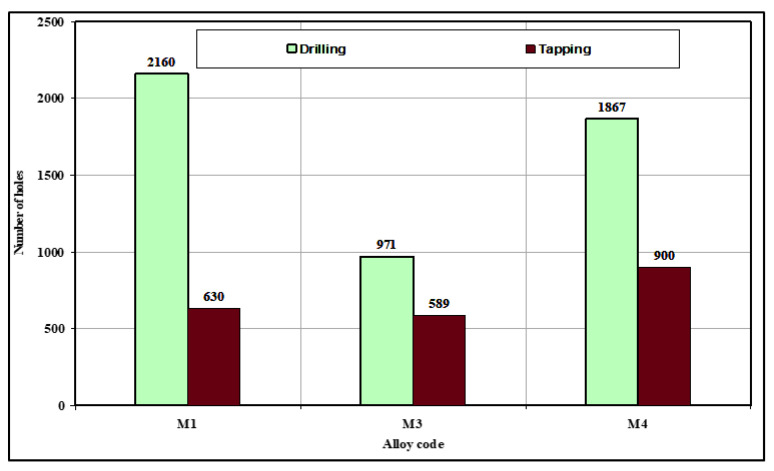
Effects of Fe-intermetallics on the drill/tap life of the M1, M3, and M4 alloys in terms of the number of holes drilled and tapped.

In tapping tests, it was found that high-speed steel tools reacted considerably more sensitively to the Fe-intermetallic phase than did the carbide tools used for drilling. The high speed steel tap (HSS-E) broke down after tapping only 630, 589, and 900 holes in the M1, M3, and M4 alloys, respectively, as shown in Figure 11.

### 6.2. Second Set of Alloys

**Figure 12:**
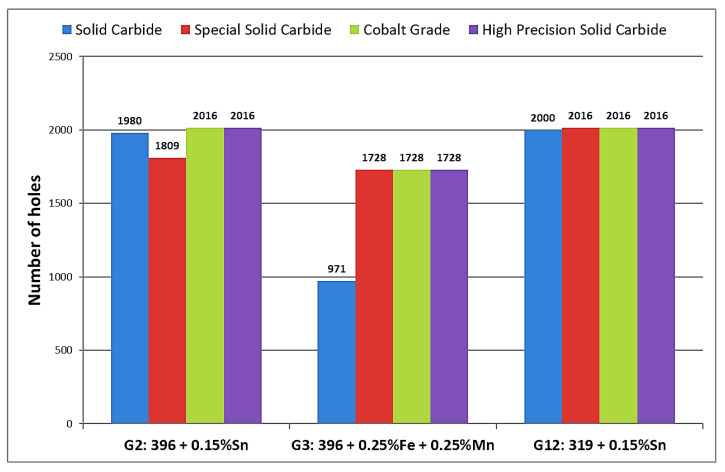
Comparison of tool life in terms of number of holes drilled for G2, G3, and G12 alloys.

Figure 12 provides the comparison between tool life in terms of number of holes drilled for each alloy used in this work. The addition of small amounts of Sn to the G2 and G12 alloys provided the desired drilling results in terms of the number of holes drilled; for both alloys, the targeted number of holes, i.e., 2016 holes, could be achieved/drilled, with the exception of when the special solid carbide drill was used, with which only 1809 holes could be drilled in the G2 alloy before drill breakdown occurred. Conversely, the drilling results obtained with the G3 alloy which contained sludge intermetallic particles, revealed that this alloy was more sensitive to tool wear than the G2 and G12 alloys. In this case, the maximum number of holes which may be drilled was found to be 1728, short of the target number by 288 holes, with the exception of the solid carbide drill which obtained only 971 drilled holes before breaking down. 

**Figure 13:**
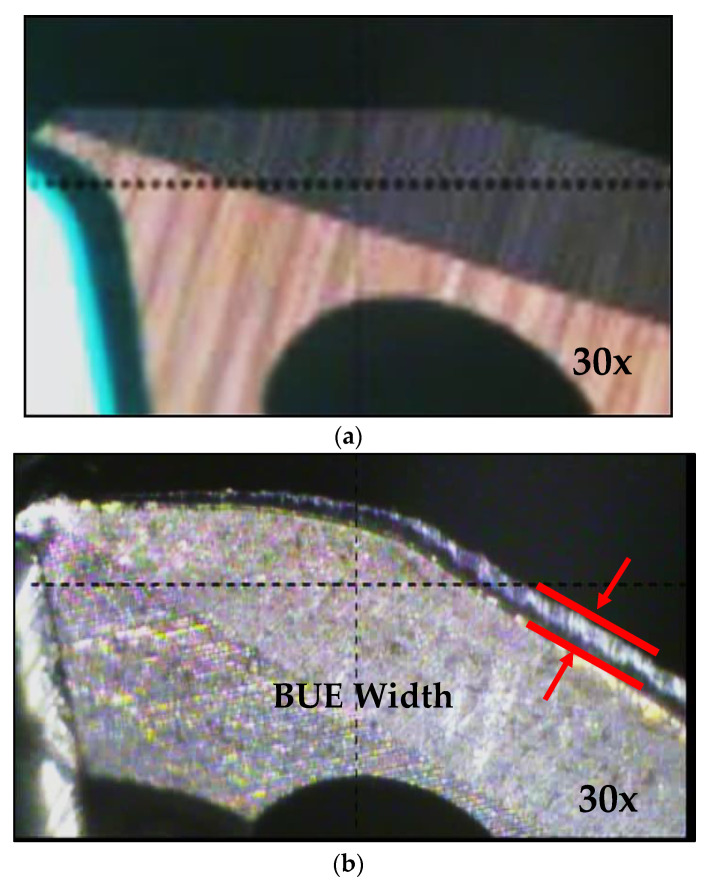
Photographs showing the effects of Fe and Mn additions on edge build-up formation and wear of the cutting drill lip in the G3 alloy after different stages of drilling using the special solid carbide drill. (**a**) Special solid carbide–new drill. (**b**) G3 alloy (BUE after 2016 holes) special solid carbide drill.

From these results, it is clear that the sludge phase, which had a high hardness value, a high melting point, and high specific gravity compared to the matrix, was capable of causing damage to cutting tools. These observations are in good agreement with the work of Zedan et al. [7], who reported that the sludge phase is liable to cause rapid tool wear and to bring on all of the attendant problems accompanying a dull tool, as was confirmed by the drill wear examination in the present study and shown in the photographs displayed in Figure 13.

## 7. Chip Formation

During drilling chips are formed when conical chips are unable to curl sufficiently to follow the flute; they tend to fracture prior to the completion of one revolution. The fan shape was by far the predominant form and is considered to be the ideal chip form for most drilling applications. The results show that the addition of iron and manganese, creating the M3 and M4 alloys, produced no discernible effect on the chip configuration compared to the 396 base alloy. It was also found that there was no clear demarcation between the needle- and fan-shaped chips. It was observed that it was the marked change in the radius of the chip about the lip axis which resulted in the needle shape; this type of radius change occurs when the build-up edge alters the geometry of the drill cutting surface.

**Figure 14:**
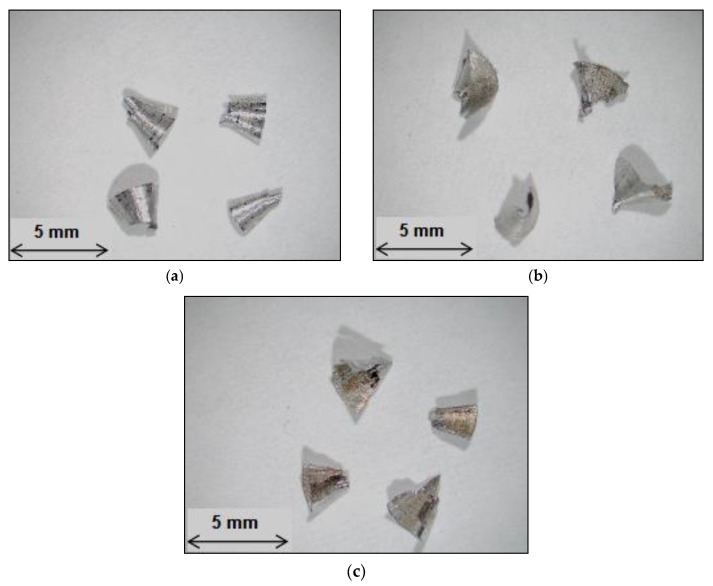
Optical micrographs showing the different types of chips obtained for (**a**) G2, (**b**) G3, and (**c**) G12 alloys after the drilling of 2016 holes using the cobalt grade drill.

The qualitative inspection of the drilling chips obtained from the second set revealed that the form of the chips resulting from the 396 and B319.2 alloys closely approached a fan-shaped form and was by far the predominant form of all the chips formed, as shown in Figure 14. These observations are in good agreement with the work of Zedan [26], who reported that these chips are formed when conical chips are unable to curl sufficiently to follow the flute, and they thus tend to fracture prior to one complete revolution of the drill; the fan shape is considered to be the ideal chip form for most drilling applications. It should be noted that free machinability is related to the formation of smaller chips. In the current study, chip size was not observed to be significantly different after the addition of free-machining elements. The results also showed that the addition of iron and manganese, creating the G3: 396 + 0.25% Fe + 0.25% Mn alloy, produced no discernible effect on the chip configuration compared to the G2: 396 + 0.15% Sn, and the G12: B319.2 + 0.15% Sn alloys, as shown in Figure 14.

## 8. Conclusions

Based on the results obtained from the present study performed on heat-treated 396, B319.2 and A356 alloys, the following conclusions can be drawn:

1. The drilling tests reveal that the 396-M1 base alloy displays a rapid increase in the mean total drilling force, moment, and power by 103%, 105%, and 134%, respectively, as the number of holes drilled increases.
2. The addition of 0.25% Fe and 0.25% Mn to the 396 base alloy, leads to an extremely rapid increase, of about 75%, in the total drilling force after drilling only 540 holes.
3. The formation of the *α*-Fe phase in the 396 alloy produces the highest number of holes drilled compared to sludge- or *β*-Fe containing alloys.
4. Increasing the iron level from ~0.5 to 1% while maintaining the same Mn level of ~0.5% proves to be most effective in terms of cutting force and tool life in the drilling and tapping processes.
5. The lower ductility value displayed by the *β*-Fe-containing alloy lessens the drill-chip friction which decreases the drilling forces, moment, and power.
6. The high precision solid carbide drill is recommended for alloys G2 (396 + 0.15% Sn) and G3 (396 + 0.25% Fe + 0.25% Mn). This drill displays stable behavior when in operation, thereby obtaining the lowest total drilling force and total drilling moment, whereas for the G12 (B319.2 + 0.15% Sn) alloy the cobalt grade drill is recommended, since it was observed to obtain the lowest total drilling force and moment.

## Figures and Tables

**Table 1:** Chemical composition of the 396 and B319.2 base alloys.

Alloy Type	Element (wt%)								
	Si	Cu	Mg	Fe	Mn	Sr	Ti	Al	Mn/Fe
396	10.89	2.243	0.309	0.464	0.492	0.014	0.057	bal.	1.06
B319.2	7.16	3.32	0.29	0.29	0.27	0.019	0.27	bal.	0.95

**Table 2:** Average chemical composition of the first set of alloys discussed in the present study.

Alloy Code	Element (% wt)													
	Si	Cu	Mg	Fe	Mn	Sr	Ti	Sn	Pb	Bi	Zn	Al	Mn/Fe	S.F ^a^
M1	11.38	2.22	0.335	0.466	0.547	0.0183	0.16	0.00	0.00	0.00	0.00	bal.	1.17	1.561
M2	11.32	2.26	0.348	0.462	0.523	0.0238	0.17	0.18	0.00	0.00	0.00	bal.	1.13	1.508
M3	11.40	2.25	0.341	0.739	0.782	0.0238	0.16	0.00	0.00	0.00	0.00	bal.	1.06	2.304
M4	11.48	2.25	0.341	0.974	0.584	0.0226	0.16	0.00	0.00	0.00	0.00	bal.	0.60	2.142

^a^: S.F.= Sludge Factor.

**Table 3:** Chemical composition of the second set of alloys investigated in the current work.

Alloy Code	Element (wt%)												
	Si	Fe	Cu	Mn	Mg	Zn	Sn	Ti	Bi	Sr	Al	Mn/Fe	S.F.
G2	10.812	0.533	2.228	0.550	0.260	0.035	0.196	0.221	0.01	0.009	84.4	1.04	1.653
G3	10.841	0.880	2.417	0.703	0.291	0.130	0.064	0.262	0.03	0.009	82.6	0.82	2.353
G12	7.533	0.346	3.597	0.286	0.240	0.040	0.178	0.241	0.00	0.009	87.3	0.83	0.934

**Table 4:** Chemical compositions for A356 and 319 alloys used for the machinability test-third series.

Alloy Code	Element (wt%)								
	Si	Fe	Mn	Mg	Cu	Ti	Sr	Mn/Fe	Al
AA356 alloy–M1	6.85	0.44	0.30	0.33	0.05	0.15	0.0218	0.69	91.7
A319 alloys–M2,	6.20	0.40	0.29	0.10	3.40	0.15	0.0234	0.73	89.3
M3	6.20	0.97	0.39	0.10	3.40	0.14	0.0236	0.40	88.6
B319.2–M4,	6.25	0.42	0.30	0.28	3.50	0.15	0.0133	0.72	88.7
M5	6.30	1.02	0.39	0.28	3.40	0.15	0.0260	0.38	88.3

**Table 5:** Summary of eutectic Si-particle measurements for the alloys studied in the T6 condition–first set of alloys.

Alloy Code	Particle Area (µm^2^)		Particle Length (µm)		Roundness Ratio (%)		Aspect Ratio		Density (Particles/mm^2^)
	Av	SD	Av	SD	Av	SD	Av	SD	
M0 *	65.70	84.10	20.80	17.10	32.80	18.20	3.51	2.32	2604
M1	4.30	6.51	2.96	2.49	70.00	17.30	1.77	0.78	28,000
M3	4.98	4.67	3.34	2.07	68.30	16.10	1.71	0.66	27,883
M4	4.89	4.62	3.49	2.02	67.10	15.80	1.70	0.89	27,022

* Base alloy–no modification or grain refiner.

**Table 6:** Summary of eutectic Si-particle measurements of the studied alloys-second set of alloys.

Alloy Code	Particle Area (μm^2^)		Particle Length (μm)		Roundness Ratio (% )		Aspect Ratio		Density (Particles/mm^2^)
	Av	SD	Av	SD	Av	SD	Av	SD	
G2	17.07	18.60	8.16	6.68	39.97	20.83	2.76	1.65	41,071
G3	9.10	12.10	4.87	4.12	51.60	20.90	2.02	1.06	67,114
G12	7.44	10.60	4.33	3.73	60.00	18.90	2.07	1.05	12,000

**Table 7:** Summary of mechanical properties for the alloys studied.

Alloy Code	BHN (MPa)	YS (MPa)	UTS (MPa)	El (%)
M0	119 ± 3.45	346.11 ± 5.92	382.78 ± 2.36	0.92 ± 0.09
M1	108 ± 3.56	358.1 ± 1.55	394.04 ± 6.27	0.98 ± 0.12
M3	111 ± 4.39	326.84 ± 2.13	354.72 ± 4.86	0.84 ± 0.04
M4	113 ± 3.95	320.4 ± 12.7	339.5 ± 9.85	0.51 ± 0.02

**Table 8:** Mechanical properties summary of T6 heat-treated alloys.

Alloy Code	BHN (MPa)	YS (MPa)	UTS (MPa)	El (%)
G2 396 + 0.15% Sn	106 ± 3.19	351.65 ± 2.57	390.54 ± 5.73	1.02 ± 0.15
G3 396 + 0.25% Fe + 0.25% Mn	111 ± 4.39	326.84 ± 2.13	354.72 ± 4.86	0.84 ± 0.04
G12 B319.2 + 0.15% Sn	108 ± 3.39	392.83 ± 3.48	404.08 ± 3.75	0.77 ± 0.03

## Data Availability

Data will be made available upon request.
